# Genotypes and drug susceptibility of Mycobacterium tuberculosis Isolates in Shihezi, Xinjiang Province, China

**DOI:** 10.1186/1756-0500-5-309

**Published:** 2012-06-19

**Authors:** Juan Zhang, Ligu Mi, Yuanzhi Wang, Peizhi Liu, Haiyan Liang, Yi Huang, Bing Lv, Li Yuan

**Affiliations:** 1 Department of Pathogenic Biology and Immunology, School of Medicine, Shi Hezi University, Shihezi, People’s Republic of China; 2 The First Hospital of Shi Hezi University, Shihezi, People’s Republic of China; 3 National Institute for Communicable Disease Control and Prevention, Chinese Center for Disease Control and Prevention, State Key Laboratory for Infectious Disease Prevention and Control, Beijing, People’s Republic of China

**Keywords:** *Mycobacterium tuberculosis*, Genotype, Drug susceptibility test, Xinjiang

## Abstract

**Background:**

Tuberculosis (TB) remains a major global health problem. To investigate the genotypes of *Mycobacterium tuberculosis* (MTB) and the distribution of Beijing family strains, molecular epidemiology technologies have been used widely.

**Methods:**

From June 2010 to June 2011, 55 *M. tuberculosis* isolates from patients with pulmonary TB were studied by Beijing family-specific PCR (detection of the deletion of region of difference 105 [RD105]), and mycobacterial interspersed repetitive units variable number tandem repeat (MIRU-VNTR) analysis. Twenty-four MIRU-VNTR loci defined the genotypes and clustering characteristics of the local strains. All strains were subjected to a drug susceptibility test (DST) by the proportion method on Lowenstein-Jensen (LJ) culture media.

**Results:**

Fifty-five clinical isolates of MTB were collected. Beijing family strains represented 85.5% of the isolates studied. Using 24 loci MIRU-VNTR typing categorized the strains into eight gene groups, 46 genotypes, and seven clusters. 83.6% (46/55) of the isolates belonged to the largest gene group. Thirty-six isolates (65.5%) were susceptible, nineteen (34.5%) were resistant to at least one drug, seven (12.8%) were Multidrug-Resistant Tuberculosis (MDR TB), and two (3.6%) were extremely drug-resistant tuberculosis (XDR-TB).

**Conclusion:**

The results showed there were obvious polymorphisms of VNTRs of MTB clinical strains. Beijing family strains of MTB were predominant in the Shihezi region of Xinjiang province. There was no correlation between the drug-resistance and Beijing family strains of MTB. It is necessary to strengthen the monitoring, treatment, and management of drug-resistance TB in Shihezi region, Xinjiang.

## Background

*Tuberculosis (TB)* is an infectious disease caused by the bacillus *Mycobacterium tuberculosis (MTB)*. *TB* remains a major public health threat worldwide. China has occupied second place, behind India, among the top five high-burden countries for the last decade (http://www.who.int/tb/en). In 2010, there were 8.8 million (range, 8.5–9.2 million) incident cases of *TB*, and 1.1 million (range, 0.9–1.2 million) deaths from TB among HIV-negative people [1]. Genotyping methods have been extensively used to analyze the recent transmission dynamics of *MTB*. Different PCR-based genotyping approaches targeting the variable number of tandem repeats (VNTR) have been developed, based on the mycobacterial interspersed repetitive units (MIRU) [2,3], which are considered a good alternative to the reference method and have proven to be faster and easier to perform. The discriminatory power of MIRU-VNTR analysis is related to the number of loci. MIRU-VNTR genotyping is performed by amplifying a panel of 12, 15, or 24 loci [4]. More recently, a set of 24 MIRU-VNTR loci was reported to have greater discriminatory power than the original 12 loci system and may exceed that of restriction fragment length polymorphism (RFLP) when combined with spoligotyping [5,6]. The MIRU-VNTR method is a reliable and reproducible typing method with high discriminatory power for studying the *MTB* population structure in different countries [7-12].

The *MTB* Beijing family, first identified in 1995 in Beijing, China, is ubiquitously and significantly prevalent in certain world regions, e.g., East Asia [13]. Members of the *MTB* Beijing family are a major concern because of their high prevalence in tuberculosis patients and their high rate of multi-drug resistance [14]. Several studies have observed that the Beijing family *MTB* strains exhibits important pathogenic features that might be associated with drug resistant *TB* in China [15-17]. As the prevalence of drug resistant clones of *MTB* varies from one area to another, studies of the geographical distribution of resistant clones are useful for understanding the epidemiological characteristics of *TB*. Spoligotyping usually determines the genotype of a Beijing strain of MTB. However, this technique requires special equipment and is time-consuming. A previous study demonstrated a high the correlation between Spoligotyping and the RD105 deletion for the identification of the Beijing family genotype [17]. The simple and rapid new method, the RDl05 deletion test, was used to identify the Beijing family instead of Spoligotyping [18]. RD105 deletions have previously been reported to be Beijing family strains-specific. One large sequence polymorphism, the (LSP)-RD105 genomic deletion, was observed in all Beijing family strains and thus serves as a useful marker for the identification of this family of strains [19]. The RD105 deletion test was performed to identify Beijing family isolates in different countries [20].

Xinjiang is a province on the Northwestern coast of China. The incidence of *TB* in Xinjiang is estimated at 463 cases per 100,000 persons per year. The prevalence of drug-resistant *TB* in Xinjiang Province is higher than the average level in China. Xinjiang is a multiethnic area, being home to 55 ethnicities. The most numerous are the Uygur (46.06%), followed by Han (39.33%), and Kazakh (7.08%). In the northern regions of Xinjiang, most of the population is Han, but in the southern regions, about 85% of the population is Uygur, and only 8% of the population is Han. Shihezi region is in northern Xinjiang and has area of 456.8 square kilometers and a population of 630,000; 95% of the population is Han. In 2008, the network epidemic of Shihezi reported 991 tuberculosis cases and prevalence of 152.15/100,000. *TB* ranks as the second statutory infectious disease in Shihezi [21].

The main goal of this study was to genotype MTB strains circulating in the Shihezi region of Xinjiang Province using MIRU-VNTR-24 locus analysis and to understand the genetic diversity of Beijing and non-Beijing isolates by the RD105 deletion test. We also sought to the determine drug susceptibility patterns of the *MTB* isolates and whether the drug resistance of epidemic *TB* are directly related to the spread of Beijing family strains.

## Methods

### Mycobacterial specimens

This study included *M. tuberculosis* samples isolated between June 2010 and June 2011 from Shihezi in Xinjiang Province. Standard questionnaires were used to collect classical epidemiology data. Information was obtained on sex, age, place of birth, recent positive smear test, previous history of *TB*, and current address.

### Strain isolation and drug susceptibility test

The sputum samples were cultured and isolated on Lowenstein-Jensen (LJ) culture media. Four first-line anti-*TB* drugs (isoniazid [INH], rifampicin [RFP], streptomycin [SM], and ethambutol [EMB]) and seven second-line anti-*TB* drugs (ofloxacin [Ofx], Capreomycin [Cm], Amikacin [Am], Kanamycin [KM], P-aminosalicylicacid [PAS], Ethionamide [Eto], and Cycloserine [Cs]) were incorporated into LJ medium, at the following concentrations: INH0, 2 μg/ml; RFP, 40.0 μg/ml; SM, 4.0 μg/ml; EMB, 2.0 μg/ml; Ofx, 2.0 μg/ml; Cm, 40.0 μg/ml; Km, 30.0 μg/ml; Am, 40.0 μg/ml; PAS, 1.0 μg/ml; Eto, 40.0 μg/ml; and Cs, 40.0 μg/m, and used to detect the drug-resistance of the *MTB* by the proportion method. Strain were scored as resistant to a specific drug, or were defined as sensitive thief their growth rate was < 1% compared to the control. Strain isolation, identification, and drug susceptibility tests (DST) were performed at the Ministry of Education Key Laboratory of Xinjiang Endemic and Ethnic Disease.

### Genomic DNA extraction and molecular identification of *M. tuberculosis* isolates

Mycobacterial genomic DNA was extracted from mycobacterial colonies growing on LJ medium. Scraped colonies were dissolved in 200-300 μl of distilled water and inactivated at 85°C for 30 min, before being centrifuged at 8000 r/min for 5 min. The pellets were resuspended in 300 μl of TE (pH 8.3), boiled for 30 min, and centrifuged at 10,000 r/min for 5 min. Supernatants were collected and stored at −20°C until further use [22].

Molecular identification of the mycobacterial isolates was performed using PCR amplification of the 16 S rRNA gene and MTP40 gene [23]. The PCR mixture consisted of 0.2 μg DNA template, 3 μl buffer, 4 μl 10 mM deoxynucleoside triphosphates, 1 μl of each primer (10 pmol/μl), and 1 μl DNA Taq polymerase. The amplification cycle was 5 min at 95°C; followed by 30 cycles of 40 s at 95°C, 50 s at 65°C, and 40 s at 72°C; with a final 10 min at 72°C. PCR products were analyzed on a 2% agarose gel against a 100-bp DNA ladder.

### Genotyping by MIRU-VNTR PCR

MIRU-VNTR genotyping was performed by amplifying the 24 MIRU-VNTR loci as described previously in a technical guide [24]. The 24 MIRU-VNTR genetic loci consisted of ten original MIRU-VNTR loci; six loci of exact tandem repeats (ETRs: ETR-A, -B, -C, -D, -E and –F), five Mtub loci (Mtub4, 21, 30, 38, and 39), and three Queen’s University of Belfast (QUBs) loci (QUB-11b, -26, and 4156c). Primers were as described by Supply et al. [25]. For each reaction, DNA from *M. tuberculosis H37Rv* was used as a positive control, and sterile water was used as a negative control. PCR products were electrophoretically separated on 2% agarose gels, using a 100-bp DNA ladder as size markers. From the gel images, the corresponding MIRU-VNTR bands were interpreted as copy numbers based on the reference table in the Supply 2005 protocol [24]. The copy number at each locus was calculated using the Quantity 1 gel imaging system.

### Beijing Family strains analysis

The identification of Beijing Family strains was performed by detecting deletions in region of difference 105 (RD105) by PCR [18]. Each PCR mixture was prepared in a volume of 15 μl containing 50 ng of DNA, 1U of Taq polymerase, a 0.2 mM concentration of each deoxynucleoside triphosphate (dNTP), and 0.2 μM (each) primer. The amplification cycle was 5 min at 94°C; followed by 30 cycles of 30 s at 94°C, 30 s at 62°C, and 45 s at 72°C; with a final step for 10 min at 72°C. PCR products were electrophoretically separated on 2% agarose gels.

## Results

### Patient characteristics

Ninety sputum specimens from patients with pulmonary *TB* were collected from June 2010 to June 2011. All of the pulmonary *TB* patients were Han Chinese. Among 90 patients, 30 (33.3%) specimens were either culture negative or had culture contamination and were excluded. In addition, another five specimens (5.5%) were excluded because their cultures were mycobacteria other than *TB (MOTT)*. Therefore, 55 culture positive specimens were used for this study.

Of the 55 patients, 20 were new *TB* cases and 35 were previously treated patients. The mean age of the patients was 47.21 yr (± 2.26). There were 38 males (69.1%) and 17 females (30.9%).

### MIRU-VNTR genotyping

**Figure 1:**
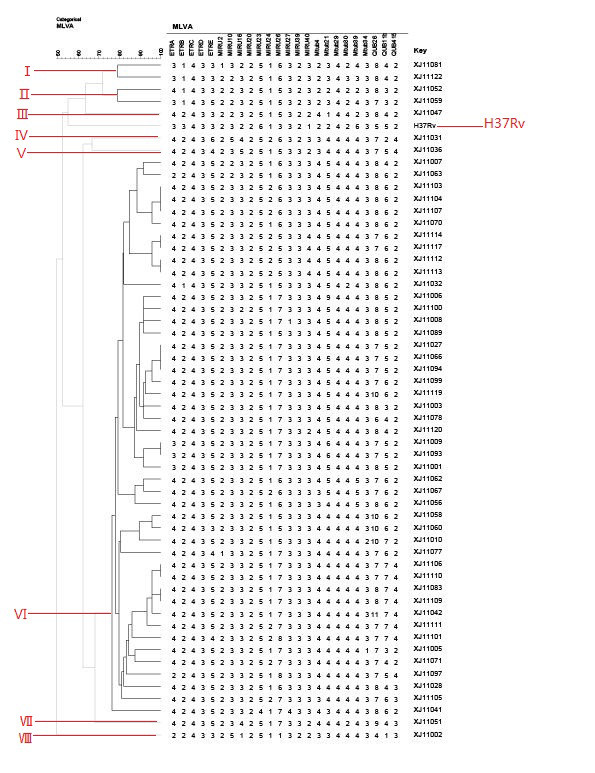
**Dendrogram deduced from clustering analysis of 55 isolates.** By using the 24 loci MIRU-VNTR typing, the results showed that 55 strains were categorized into 8 gene groups, 46 genotypes and 7 clusters.83.6% (46/55) isolates belonged to the largest gene group.

Fifty-five *MTB* isolates were genotyped and 46 different VNTR genotypes were detected. Thirty nine strains (70.9%) were unique and 16 strains (29.1%) could be grouped into seven clusters, each including 2–3 strains (Figure 1). Two main clusters which contained 3 (18.7%) and 3 (18.7%) strains showed 4 2 4 3 5 2 3 3 2 5 2 6 3 3 3 4 5 4 4 4 3 8 6 2 and 4 2 4 3 5 2 3 3 2 5 1 7 3 3 3 4 5 4 4 4 3 7 5 2 VNTR profiles, respectively. The isolates were divided into eight groups based on phylogenetic clustering and genotypic characteristics. Groups I to VIII contained 2, 2, 1,1,1,46,1 and 1 isolates, respectively (Table 1). All the clustered isolates were in group VI. In this study, H37Rv separated into a group (Figure 1).

**Table 1:** MTB characteristics in the eight subgroups

**Isolate characteristic**	**Total no. of isolates**	**No. (%) of isolates by subgroup**								**P value**
		I (n = 2)	II(n = 2)	III (n = 1)	IV (n = 1)	V (n = 1)	VI (n-46)	VII (n = 1)	VIII (n = 1)	
Beijing strain	47	0	0	0	0	1	45	1	0	<0.0001
Clustered	16	0	0	0	0	0	16	0	0	0.731

n, number of isolates in the subgroup.

### Epidemic of Beijing family strains in Shihezi of Xinjiang Province

During the study period, 55 *M. tuberculosis* isolates were identified using molecular methods. We used the RD105 deletion test instead of spoligotyping, and found that 47 of the 55 isolates (85.5%) were of the Beijing family genotype, while eight (24.5%) were non-Beijing family strains.

### Characteristics of the clustered isolates

Among the 47 Beijing family isolates, 45 (95.7%) were in group VI and the remaining two isolates were in groups V and VII. This suggested that the Beijing family isolates distributed mainly in group VI. Sixteen isolates (16/47) were clusters, and were all Beijing family isolates. In contrast, none of the eight non-Beijing family isolates were clustered (Table 2).

**Table 2:** MTB characteristics between Beijing and non-Beijing family strains

**Characteristic**	**Total no. of** **isolates (n = 55)**	**No. of isolates**		**P value**
		**Beijing (n = 47) non-Beijing (n = 8)**		
Resistance				
INH	8	7	1	
RFP	5	4	1	
SM	12	11	1	
EMB	6	6	0	
PAS	3	3	0	
KM	1	1	0	
Eto	2	2	0	
Cs	2	2	0	
CM	2	1	1	
Am	1	1	0	
Ofx	4	3	1	
Any drug-resistance	19	17	2	0.429*
MDR	7	5	2	0.386*
XDR	2	2	0	1*
Susceptibility	36	30	6	0.429*
Clustered	16	16	0	0.113*

*Value by Fisher’s exact test.

### Drug susceptibility patterns of the MTB isolates

The 55 *MTB* strains isolated from the sputum samples of *TB* patients were subjected to a drug susceptibility test. Drug susceptibility with the four first-line anti-tuberculosis and the seven second-line anti-tuberculosis drugs was examined to determine the association between drug resistance patterns and genotype.

Thirty-six isolates (65.5%) were susceptible to all eleven drugs; 19 (34.5%) were resistant to at least one drug; seven (12.8%) were *MDR-TB* strains, which were resistant to at least INH and RIF, the two most powerful anti- tuberculosis drugs; two (3.6%) were *XDR-TB*, defined as *MDR TB* with further resistance for any quinolones and to >1 of the three classes of second-line drugs (Cm, Am, or Km)[26]. We examined the distribution of drug resistance between Beijing and non-Beijing strains. Among the seven *MDR-TB* isolates, five (10.6%, 5/47) isolates were Beijing family strains, and two (0.25%,2/8) were non-Beijing family strains. The rates of *MDR-TB* among Beijing and non-Beijing family strains were not statistically different (P = 0.386). Among the Beijing family strains, 36.2% (n = 17) were resistant to any drug; among the non-Beijing family strains, 2.5% (n = 2) were resistant to any drug (Table 2).

Of the 55 patients, 20 (36.4%) were new *TB* cases and 35 (63.6%) were previously treated patients. Among the four *MDR-TB* patients, all had been previously treated, while among the 51 patients with non-*MDR-TB*, 31 patients (60.8%) had been previously treated. However, there was no association of *MDR-TB* cases with either new cases or previously treated cases, as shown in Table 3.

**Table 3:** MTB characteristics between new cases and previously treated cases

**Type**	**Total** **n = 55**	**New cases** **n = 20**	**Previously treated cases n = 35**	**P value**
Susceptible	36	12	24	0.361*
Any drug-resistance	19	8	11	0.361*
MDR	7	0	7	0.154*
XDR	2	0	2	0.264*
Beijing family	47	16	31	0.216*
Clustered	16	5	11	0.427*

*Value by Fisher’s exact test.

## Discussion

Recent advances in molecular technology, such as RD105 deletion and MIRU-VNTR typing, have provided powerful tools for analyzing *MTB* genotype and transmission patterns, and should prove invaluable for developing effective infection-control policies.

The RDl05 deletions, which can identify the Beijing Family strains, are very valuable molecular markers. In this study, we found that 85.5% of *MTB* isolates in the Shihezi region were Beijing family strains. In the previous study, the prevalence of Beijing family strains were different in various regions of China, e.g., 80% to 90% in Beijing, 67% in Ningxia, 89% in Shanghai, 70% in Zhejiang, 91.5% in Tianjin, 55.3% in Guangxi, 80.4% in Jiangsu, and 89.5% in Heilongjiang [12]. Thus, the Beijing family strains were prevalent in China. The association of drug resistance and the Beijing family strains has become a research hotspot. However, results obtained differ. These strains may have a particular propensity for acquiring drug resistance [27]. In our study, the statistical analysis showed that there was no difference between the Beijing and non-Beijing family strains in terms of their drug resistance patterns, indicating that the Beijing family was less likely to be associated with the high prevalence of drug resistance in Shihezi region.

In this study, general resistance to at least one drug and MDR were all higher than the resistance rate observed in 2008 [28]. The presence of two *XDR-TB* cases (3.6%) was another important discovery, and it is the first report of *XDR-TB* cases in Xinjiang. Worldwide, *XDR-TB* cases have been reported in 45 countries [29]. In China, a survey of resistance to tuberculosis showed that the rate of *XDR-TB* was 0.68% in 2007–2008 [30]. Therefore, we should pay more attention to *MDR-TB* and *XDR-TB* to control tuberculosis.

The MIRU-VNTR method has been used in molecular epidemiology studies, and it is adequate for tracking recent transmission and distinguishing relapses and reinfections [9]. In addition to its high discriminatory power, MIRU typing is simple to perform, has a high throughput, and is highly reproducible with a short turnaround time. MIRU types are represented in digital format; therefore, results from different laboratories can be easily compared. In this study, 55 isolates were classified into 46 types, eight gene groups, and seven clusters by MIRU-VNTR typing. The eight major gene groups, combined with flow epidemiological data, did not identify the means of mutual transmission and direct contact, because we only focused on strains collected in June 2010 to June 2011. Patients of the experimental investigation distributed in a different location of the transmission chain, and were insufficient to reflect the full spread relationship. At the same time, we were unable to collect the strains of previously treated patients; therefore, the infection pattern may be the recurrence of endogenous or recent infection. The analysis indicated that the Beijing family isolates were less likely to be part of a cluster. Future studies using more isolates are required to confirm this hypothesis.

## Conclusion

This is the first report of the genotypes of *MTB* isolated from patients with pulmonary TB in the Shihezi region of Xinjiang Province, China. We defined 24 MIRU-VNTR loci for analyzing the strains. The 55 isolates show a high number (16/55) of clusters, and Beijing family strains (47/55) are prevalent in Shihezi region. The drug-resistance rate was high; in particular, two *XDR-TB* cases were found. It is necessary to strengthen the monitoring, treatment and management for drug-resistance *TB*. There was no correlation between drug-resistance and the Beijing family genotype.

## Authors’ contribution

LY designed and supervised experiments, and collected the sputa samples; JZ wrote the manuscript, collected the sputa samples, and cultured and identified all strains; JZ, LG Mi, and PZL performed the VNTR genotyping of MTB; YZ. Wang identified the Beijing family strains; HYL and YH performed the drug susceptibility tests; and BL analyzed the data. All authors read and approved the final manuscript.

## Competing interests

The authors declare that they have no competing interests.
